# Synthesis and Study of the Optical Properties of PMMA Microspheres and Opals

**DOI:** 10.3390/polym13132171

**Published:** 2021-06-30

**Authors:** Mayra Matamoros-Ambrocio, Enrique Sánchez-Mora, Estela Gómez-Barojas, José Alberto Luna-López

**Affiliations:** 1 Centro de Investigación en Dispositivos Semiconductores, Instituto de Ciencias, Benemérita Universidad Autónoma de Puebla, P.O. Box 196, 72000 Puebla, Mexico; mayra.matamoros@alumno.buap.mx (M.M.-A.); egomez@ifuap.buap.mx (E.G.-B.); jose.luna@correo.buap.mx (J.A.L.-L.); 2 Instituto de Física, Benemérita Universidad Autónoma de Puebla, Eco campus Valsequillo, Independencia O 2 sur No. 50, San Pedro Zacachimalpa, P.O. Box J-48, 72960 Puebla, Mexico

**Keywords:** PMMA microspheres, opals photonics crystals, optical properties

## Abstract

Polymethylmethacrylate (PMMA) microspheres were synthesized by surfactant-free emulsion polymerization. These microspheres were used to obtain opals by the self-assembly method. Monomer and initiator quantities were varied systematically to monitor the size of PMMA microspheres. From SEM and DLS measurements, a trend was observed showing as the monomer and initiator amounts increased the average diameter of PMMA microspheres increased except when a minimum monomer amount was reached, for which the size of the microspheres remained practically constant. Diffuse reflectance spectra were processed by the Kubelka–Munk treatment to estimate the energy band gap (E_g_) of the PMMA microspheres. It was found that PMMA microspheres present an indirect transition. From SEM micrographs, it is seen that PMMA opals photonic crystals are formed by microspheres in a uniform periodic face-centered cubic (fcc) array. Variable-angle specular reflectance spectra show that the opals possess a pseudo photonic band gap (PBG) in the visible and near-IR regions. Furthermore, it was found that PBGs shift towards larger wavelengths as the average diameter of the PMMA microspheres increases.

## 1. Introduction

Photonic crystals are ordered structures whose dielectric constant is modulated with periods on the wavelength scale of visible light [1]. In nature, they are present in some butterfly wings, bird feathers, and opal gemstones. In these structures, periodicity in the refraction index gives rise to diffraction effects of photons on dielectric lattice planes, resulting in a photonic band gap (PBG) or a stop band that inhibits a range of wavelengths from propagating through the crystal [2]. 

The photonic crystals with complete photonic band gaps have been studied with great interest because of their applications in different areas of science and technology. For example, optical sensors have been coupled to a transduction device that identifies the optical diffraction that occurs once the external medium changes [3]. Optical waveguides [2], switches [4], light-emitting diodes [5], and templates to fabricate devices with interconnected porous matrices have also all been examined [6,7]. In order for the gamma of applications to be successful, improved methods for the fabrication of highly ordered 3D photonic crystals must be developed.

Opals are distinguished as a class of photonic crystals mainly obtained by a self-assembly approach, where particles are organized spontaneously to form ordered three-dimensional structures [8]. The most common methods used are: gravity sedimentation, centrifugation, vertical deposition, and spin coating [9]. The materials most widely used for opals are silica [10] and polymers as PS [11] and PMMA [12]. On the other hand, block copolymers (BCPs) provide another route for creating photonic crystal structures, where self-assembly of high-molecular-weight block copolymers yields periodic photonic crystal. For example, polystyrene–polyisoprene diblock copolymer demonstrates that block copolymers can form self-assembly optical structures for the construction of lightweight and flexible photonic devices [13]. Additionally, the system polystyrene-b-quaternized poly (2-vinyl pyridine) (PS-b-QP2VP) lamellar films provide photonic gels with exceptionally large stop-band tunability across the ultraviolet-visible and near-infrared regions [14]. This expected system will lead to many novel applications including colorimetric sensors, active components of simple display devices, electrically controlled tunable optically pumped lasers, photonic switches, and multiband filters. Regarding the recent use of new polymeric materials, inspired by structural coloration arising from assembled melanosomes in avian feathers, they have been prepared and assembled using synthetic melanin nanoparticles that offer numerous new opportunities towards multifunctional photonics [15]. 

The opals obtained using polymer monodisperse particles have proved to be an efficient approach because the preparative process is simple, easy, and cheap [11]. Among the polymers, the most studied is PMMA because it has good thermal stability, interesting optical properties, and a high Young’s modulus of around 3 MPa, making it thermoplastic with high resistance to scratching [16].

The PMMA synthesis method most used is the surfactant-free emulsion polymerization (SFEP) because the obtained colloidal spheres have good monodispersity and clean surface features [12]. In this process, the monomer is dispersed in water and, in the presence of a free radical initiator, the polymerization is started. The oligomers act as surfactants and form micelles as soon as their concentration exceeds critical micellar concentration. The polymerization reaction proceeds within micelles analogously to the classical emulsion polymerization. The stability of the colloidal dispersion is reached due to stabilizing groups originating from the initiator molecules that are covalently bonded to the surface of spheres [17].

Using the SFEP method, synthesis conditions including temperature, stirring speed, and amounts of monomer and initiator have been proven to be key factors influencing particle size. In general, if we want to obtain small-size particles, we should increase the temperature, use a small amount of initiator, or decrease the amount of monomer. In the other case, to obtain a large particle size, the amount of monomer must be increased, the amount of initiator must be decreased, or the reaction must be carried out at low temperature [1,6,18,19,20,21]. Although these results are well established in the literature, a careful review of the synthesis of PMMA and other polymers using the SFEP method shows inconsistencies when the amount of initiator is varied [6]. Thus, in this research work we investigate the effect of the amount of monomer and the amount of initiator used to obtain PMMA microspheres by the SFEP method.

On the other hand, the optical properties of PMMA material have been widely studied and reported [22,23,24]. Although a relationship among optical properties, morphology, and size of PMMA microspheres has not been elucidated, it has been widely accepted in the literature to report the calculation of both direct and indirect energy band gaps from UV-Vis spectra. By the same token, many of the reported studies do not justify with certainty the type of transition that takes place in this material [25].

The success of making opals with good reproducibility is highly dependent on the ability to preparate monodisperse colloidal spheres with controlled size on the optimization of a synthesis reaction and experimental conditions so that the spheres self-assemble into ordered matrices with a defined structure and good reproducibility. 

The aim of this work is to study the effect on the variation of the monomer and the initiator quantity on the size of the PMMA microspheres that constitute the opals. Furthermore, the PMMA opals’ photonic crystals are characterized to determine the influence of size on their optical properties. The results can provide insight into the properties of PMMA. 

## 2. Materials and Methods

### 2.1. Materials

Methylmetacrylate (MMA; C_5_H_8_O_2_; 99%) and 2,2′-azobis (2-methylpropionamidine) dihydrochloride (V50, C_8_H_20_Cl_2_N_6_; 97%) were purchased from Sigma-Aldrich (St. Louis, MO, USA) and used without further purification. Isopropyl alcohol (C_3_H_8_O; 99%) was purchased from J. T. Baker (Phillipsburg, NJ, USA). Deionized water (DI; 18.2 MΩ cm; Thermo Fisher Scientific, Marietta, OH, USA) was used in all experiments.

### 2.2. Synthesis of PMMA Microspheres

PMMA microspheres were synthesized by means of surfactant-free emulsion polymerization method reported by E. Sánchez et al. [26]. Briefly, in a three-necked round bottom flask (250 mL) equipped with a reflux system, 160 mL of DI water and methylmethacrylate were added, and the system was kept under vigorous magnetic stirring (~400 rpm) and constant N_2_ bubbling (0.2 cm^3^ s^−1^) flux. At this moment, the temperature in the system was raised to 75 °C, then the V50 was added. Since the polymerization is an exothermic reaction, the temperature of the system reached 95 °C; this temperature was kept constant for 2 h. Finally, the reaction was allowed to cool to room temperature and the rising procedure of the PMMA microsphere was carried out by centrifugation at 6000 rpm (HERMLE Z36 HK) for 30 min. Then, the supernatant was taken away by decantation and the solid was redispersed in a solution of DI water and isopropyl alcohol (50/50 *v*/*v*) in ultrasonic for 15 min. This procedure was repeated 3 times. In total, a set of 9 samples was prepared with different monomer and initiator amounts, as summarized in Table 1.

### 2.3. Preparation of PMMA Opals

PMMA opals were obtained by adding 50 mL of the colloidal suspension in Petri dishes and left alone at room temperature (25 °C) for 3 weeks to evaporate the DI water and isopropyl alcohol in which they were dispersed. The thickness of opals was about 1 mm.

### 2.4. Materials Characterization 

The PMMA microspheres were characterized by Fourier Transform Infrared Spectroscopy (FT-IR; Spectrum One, Perkin Elmer, Waltham, MA, USA) in transmittance mode with an ATR accessory and a scanning velocity of 8 nm/s, at room temperature in the range of 650–4000 cm^−1^. Micro-Raman spectroscopy (µ-RS; LabRAM HR-Olympus, Horiba Jobin Yvon Inc. Edison, NJ, USA) with a He-Ne laser (λ = 632.8 nm) was used as an excitation source, and an optical microscope at 10× was used to select the region of the interest. The spectra were recorded from 200–2000 cm^−1^ range.

The surface morphology of both PMMA microspheres and opals was analyzed with a scanning electron microscope system (SEM; JSM-7800F, JEOL, Tokyo, Japan). The colloidal suspensions diluted in DI water were deposited on clean Si wafers. Once the solvent was evaporated from the wafers, the PMMA microspheres were observed under the SEM, and their average diameter was determined from the SEM micrographs considering about 300 spheres and using the ImageJ software.

The optical characterization of the PMMA microspheres was performed using a spectrometer UV-Vis-NIR (200–2500 nm) (Cary 5000 UV-Vis-NIR system, Aligent, Santa Clara, CA, USA) equipped with a DRA-CA-30I and a Labsphere as a reference to record diffuse reflectance spectra (DRS). The powder samples were compacted, and each sample was set on the sample holder. Additionally, it was equipped with a variable angle specular reflectance accessory (VASRA) and an Al mirror as a reference to measure the specular reflectance at different angles in the range of 20–70° of the PMMA opals. The sample was approximately 1 cm^2^ in area and it was set on the sample holder. 

The hydrodynamic diameters (D_h_) and the polydispersity indices (PDI) were measured from the PMMA dilute aqueous solutions with a dynamic light scattering system (DLS; Malvern Zetasizer Nano ZS, Malvern Instruments Worcestershire, UK) equipped with an He-Ne laser (λ=633 nm) with 4.0 mW power and backscattering mode. The PDI data were obtained directly from the equipment software. Three consecutive measurements at 25 °C were taken, and each of them consisted of 10 individual runs. The reported values are average values. The zeta potential measurements were carried out with the Zeta-Sizer IV system (Malvern Instruments Worcestershire, UK) under the same conditions as the ones used with the DLS system.

## 3. Results and Discussion 

### 3.1. PMMA Microspheres

#### 3.1.1. FT-IR and Micro-Raman Analysis

**Figure 1:**
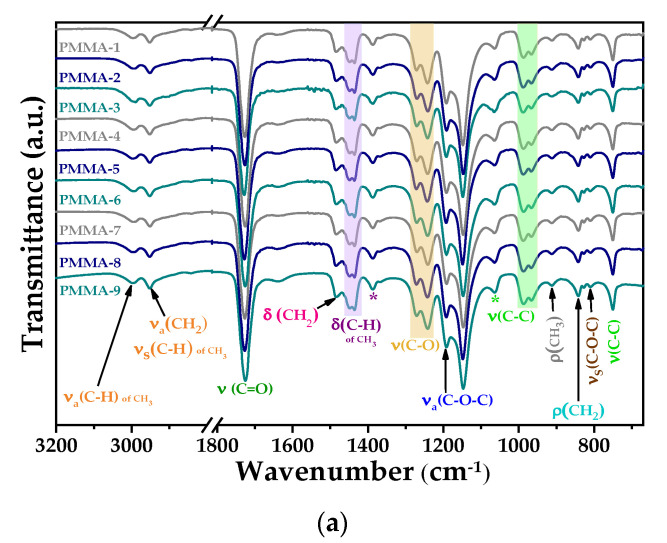

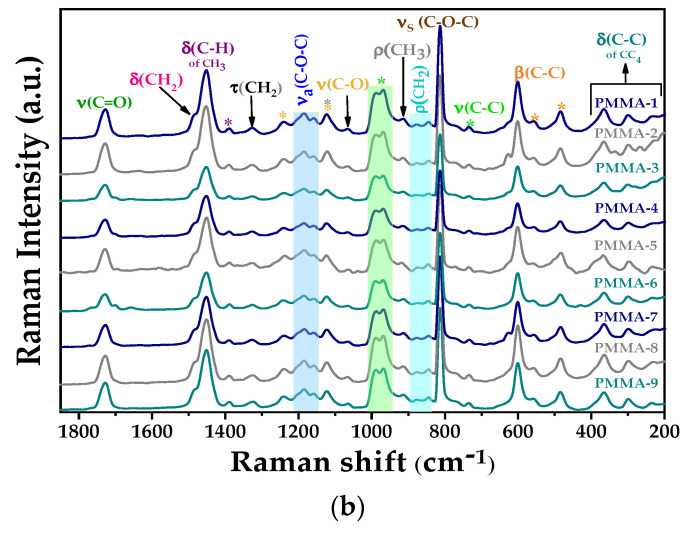
(**a**) FT-IR spectra and (**b**) micro-Raman spectra of the whole set of synthesized PMMA microspheres.

The success of the PMMA microspheres’ synthesis was confirmed by means of the FT-IR and Raman spectra shown in Figure 1. The PMMA structure contains an ester group, one methylene (CH_2_) group, and two methyl (CH_3_) groups. Therefore, the bands present in the spectra correspond to vibrational modes of these groups.

The bands at 2999 and 2952 cm^−1^ in the FT-IR spectra are assigned to symmetrical and asymmetrical stretching vibration modes of the CH_3_ group. The bands at 1448, 1436, and 1387 cm^−1^ present in the FT-IR spectrum, and the ones at 1452 and 1390 cm^−1^ in the Raman spectra, are assigned to the deformation modes of the C-H bond of the CH_3_ group. In the case of the bands at 1123 and the one at 914 cm^−1^ in the Raman spectrum, and also the one at 912 cm^−1^ in the FT-IR spectrum, these are assigned to the rocking modes of the CH_3_ groups.

The CH_2_ group also presents a symmetric stretching vibrational mode at 2952 cm^−1^ and deformation modes at 1484 cm^−1^ in the FT-IR spectrum, and at 1481 cm^−1^ in the Raman spectrum. The band at 1326 cm^−1^ appearing in the Raman spectrum is assigned to the twisting mode of the C-H bond. Finally, the rocking vibrational mode corresponds to the bands at 877 and 875 cm^−1^ present in the Raman spectrum, and to the one at 842 cm^−1^ in the FT-IR spectrum [27]. The band at 1727 cm^−1^ in the FT-IR spectrum and the one at 1728 cm^−1^ in the Raman spectrum correspond to the C=O stretching mode. For the C-O bond, the stretching modes are located in the 1000 to 1400 cm^−1^ range in both FT-IR and micro-Raman spectra, even though the bands appearing in the 900 to 1000 cm^−1^ range in both spectra bend vibrational modes of the C-C bond are observed at 484, 558, and 601 cm^−1^ of the Raman spectrum.

The bands in the range 400 to 200 cm^−1^ of the Raman spectrum correspond to the deformation modes of the C-C bonds. The remaining bands are assigned to symmetrical and asymmetrical stretching vibration modes of the C-O-C bond located at 813, 1159, and 1185 cm^−1^ of the Raman spectrum, while they are observed at 810, 1242 and 1271 cm^−1^ in the FT-IR spectrum [28]. 

#### 3.1.2. SEM and DLS Analysis

**Figure 2:**
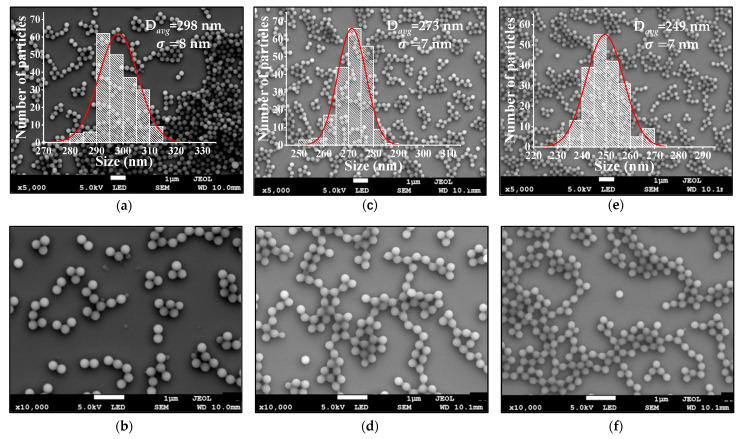
SEM micrographs of PMMA microspheres showing the formation with 285 mmol of monomer and: (**a**,**b**) 1.65 mmol of initiator, (**c**,**d**) 1.10 mmol of initiator, and (**e**,**f**) 0.55 mmol of initiator. The insets present the size distribution histograms of the PMMA microspheres of each sample.

PMMA microspheres were prepared, keeping the amount of monomer fixed at 285 mmol while varying the amounts of initiator at 0.55, 1.10, and 1.65 mmol. Figure 2 shows SEM micrographs at ×5000 of these prepared PMMA spheres. A corresponding size distribution histogram appears as an inset in each micrograph. Thus, 0.55 mmol of initiator gives PMMA spheres had average diameters (D_avg_) of 249 nm (σ = 7 nm) for 1.10 mmol, D_avg_ = 273 nm (σ = 7 nm) and, for 1.65 mmol of initiator, D_avg_ = 298 nm (σ = 7 nm). Thus, based on these SEM micrographs, we infer that the influence of the initiator is found on the size of the PMMA microspheres; moreover, the bigger the amount of initiator, the greater the diameter of the spheres. In SEM micrographs at ×10,000 in (b), (d), and (f), it is observed with more detail that all PMMA particles have a spherical shape. This result confirms the spherical term as it has been used in the text. 

The PMMA microspheres were also analyzed by means of a DLS system. The hydrodynamic diameter (D_h_) values are listed in Table 1. It is found that the PMMA microsphere diameters measured by DLS are greater than the ones given by the distribution histograms in the SEM micrographs (Figure 2). This difference is because the DLS measurements were carried out with PMMA microspheres in a water solution. Then, the formation of a water molecular layer on the PMMA microspheres’ surface must be considered. The formation of this layer is due to hydrogen bridge bonds or the PMMA microspheres’ surface protonation, which gives rise to a positive surface charge. The corresponding polydisperse indices (PDI) are also listed in Table 1. In all cases, the PDI values are less than 0.1, indicating a uniform distribution of the microspheres [29].

**Figure 3:**
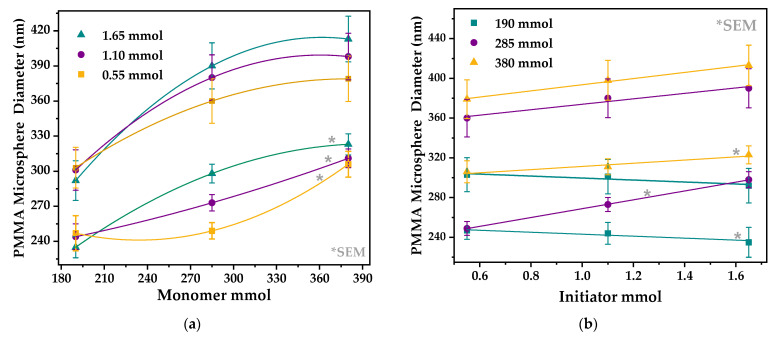
(**a**) A plot of microsphere diameter vs. MMA mmol and (**b**) Microsphere diameter vs. initiator mmol. The diameters were measured by SEM (*) and DLS techniques.

For better visualization, data in Table 1 are plotted in Figure 3a shows the diameter of the PMMA microspheres vs. the amount of monomer at a constant quantity of initiator. It is seen a nonlinear tendency of the PMMA microsphere diameters as the mmols of monomer are increased; this tendency is followed by the SEM and DLS diameters’ measurements. This behavior is in accord to the results obtained by Waterhouse et al. [1], Nandiyanto et al. [6], and Yohanala et al. [21], even when they synthesized PS (Table 2). In (b), it is seen that, for fixed amounts of monomer (285 and 380 mmol) with varying amount of the initiator (0.55, 1.10, and 1.65 mmol), the PMMA microspheres’ diameter increases linearly. However, when the monomer amount is fixed at 190 mmol with varying amounts of initiator (0.55, 1.10, and 1.65 mmol), the PMMA microspheres’ diameter decreases linearly. This result is unexpected, even though it is in accord with the tendencies reported by Yamamoto et al. [20] who, for monomer amounts fixed at 28.3 and 14.2 g and with varying amounts of initiator (0.034, 0.061, and 0.186 g), the microspheres D_avg_ tended to increase. However, for a fixed monomer amount of 2.83 g with the same increasing amounts of initiator, D_avg_ tends to decrease. This unexpected result is in disagreement with that reported by Waterhouse et al. [1], probably because they varied two parameters simultaneously: the amount of monomer and temperature (Table 2). In this regard, we emphasize that our experiments are more reliable than the ones by Waterhouse et al. [1], since we varied one synthesis parameter at a time.

**Figure 4:**
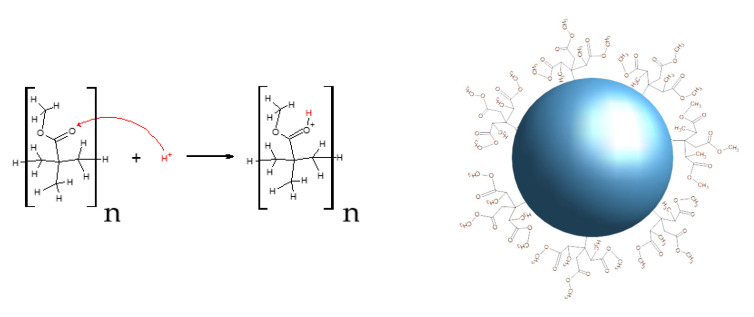
The schematic protonation reaction of the carboxyl group that surrounds the PMMA microsphere surface.

In Table 1, the ζ potential values obtained from microspheres of PMMA are listed; they lie in the 41.4 to 51.1 mV range. Therefore, it is concluded that the dispersions are very stable [29]. It is also important to mention that no relation was found between the reaction variables used in this study. Yamamoto and Higashitani [30] have suggested that a positive ζ potential for the PMMA microspheres is associated to the functional groups in the initiator V50. If this were the case, the bands corresponding to C-N and N-H vibrations would be present in the FT-IR and Raman spectra. Since, in the corresponding spectra of our microspheres set, these bands are not present (Figure 1), we infer that the ζ potential is instead positive due to the protonation of the carboxyl group, as is illustrated schematically in Figure 4 and confirmed with the pH = 3.30 measured from the colloidal PMMA microspheres. 

#### 3.1.3. DRS Analysis

**Figure 5:**
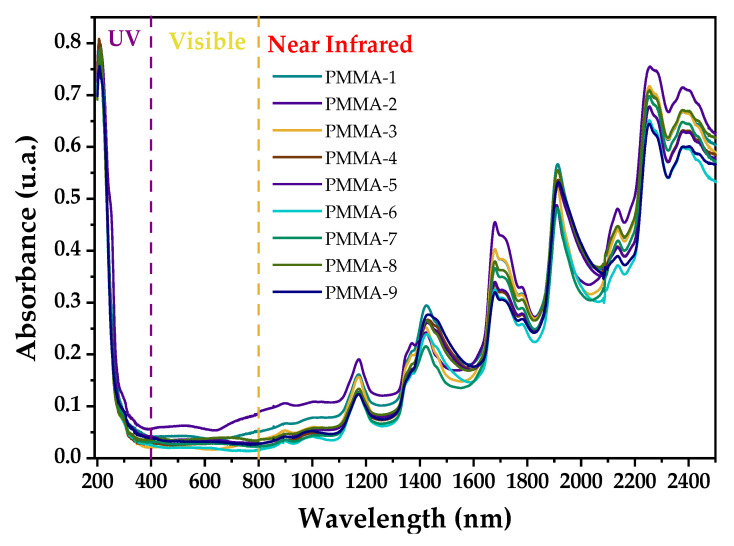
UV-Vis-NIR diffuse reflectance spectra (shown as absorbance) of PMMA microspheres.

Figure 5 shows diffuse reflectance spectra (expressed as absorbance) in the UV-Vis-NIR range of the PMMA microspheres in powder form, synthesized with different reaction conditions. All spectra in the UV region (200 to 400 nm) show a pronounced absorbance edge while, in the Vis region (400 to 800 nm), the absorption is small and almost constant. In the NIR region (800 to 2500 nm) seven bands related to the vibrations of the C-H and C-O bonds are present. A detailed analysis of the bands in the NIR region is described below.

In the NIR region, the more intense bands are located at 1173, 1425, 1679, 1912, 2136, 2256, and 2377 nm. The band at 1173 nm is assigned to the second harmonics of the fundamental stretching vibrations of the C-H, CH_3_ and CH_2_ bonds. In this region (800 to 1350 nm), the bands are of low intensity compared to the bands located at longer wavelengths due to the characteristic harmonics of the involved vibrations [31]. The CH_3_ and CH_2_ groups are also responsible for the band located at 1425 nm, which is due to combined stretching vibrations and deformation of the C-H bond. The bands at 1679 and 1697 nm are assigned to fundamental stretching vibrations of the C-H bond of the CH_3_ group, while the bands at 1717 and 1779 nm correspond to the first harmonic of the CH_2_ group [32]. The band located at 1912 has been assigned to the second overtone of the stretching of the C=O bond, while the band located at 2133 nm is due to a combination of stretching vibrations of the C-H bond of the OCH_3_ groups and to the stretching of the C=O of the ester [31]. Finally, the bands located at 2256 and 2377 nm are due to a combination of the stretching and flexing vibrations of the C-H bond in the CH_3_ and CH_2_ groups, respectively [33]. These results confirm that the C-N bonds are absent due to the initiator and the positive charge indicated in the results of the ζ potential, which is itself due to the protonation of the carboxyl group.

In the UV region, we can see a sharp absorption edge of about 270 nm due to electronic transitions from **n** level orbital (HOMO, highest occupied molecular orbital) to $\sigma^{*}$ level (LUMO, lowest unoccupied molecular orbital), according to the molecular orbitals theory [25]. This absorption edge is also associated with the energy band gap that is the difference in energy between the minimum of the conduction band and the maximum of the valence band. The E_g_ values of the PMMA microspheres were determined using the Kubelka–Munk (K-M) formalism [34] given by Equation (1):

(1) $${\left[F\left(R_{\infty }\right)hv\right]}^{1/n}=C\left(hv-E_{g}\right),$$

where h is the Planck constant, ν is the electromagnetic radiation frequency, C is a proportionality constant, and the n value depends on the nature of the electronic transition of the material. In the case of a compound with a direct energy band gap, n=2, and for an indirect energy band gap, n=1/2. $F\left(R_{\infty }\right)$ is the the K–M function, which relates the dispersion and absorption coefficients given by Equation (2):

(2) $$F\left(R_{\infty }\right)=\frac{{\left(1-R_{\infty }\right)}^{2}}{2R_{\infty }}=\frac{K}{S}$$

where $R_{\infty }$ is the ratio between the reflectance of the sample to that of the reference:

(3) $$R_{\infty }=\frac{R_{sample}}{R_{reference}}$$

It is widely accepted in the literature to determine the E_g_ values of PMMA considering both cases of direct and an indirect- band gap energy. 

**Figure 6:**
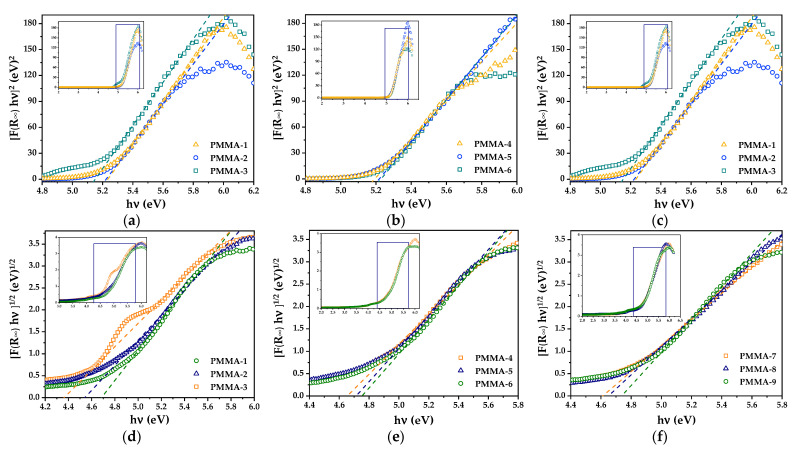
Kubelka–Munk transform reflectance spectra of PMMA microspheres in powder: (**a**–**c**) considering a direct band structure, and (**d**–**f**) considering an indirect band structure. From these spectra, the E_g_ values of the PMMA were determined.

The E_g_ values were determined by plotting ${\left[F\left(R_{\infty }\right)h\nu \right]}^{1/n}$ vs. hν, making a linear fit to the absorption edge, and the E_g_ value was obtained by the intersection to the hν axis. Figure 6 shows an ${\left[F\left(R_{\infty }\right)h\nu \right]}^{1/n}$ vs. hν plot considering in (a–c) a direct transition, and in (d–f) an indirect transition is considered. The obtained E_g_ values are listed in Table 2.

In Table 3, it is seen that the E_g_ values for a direct energy band gap are greater than those of an indirect band structure. These values are close to those of Aziz et al. [25], who reported 5.04 eV for a direct energy band gap and 4.8 eV for an indirect one in relation to PMMA thin films. However, these values are smaller than the 7.0996 eV theoretical value calculated by Hazim et al. [35] using the density functional theory. The difference in PMMA E_g_ values is due to fact that PMMA microspheres are porous and present punctual defects in their structures as grain frontiers, oxygen vacancies, etc., which are the same as those presented by amorphous SiO_2_ microspheres [10].

**Figure 7:**
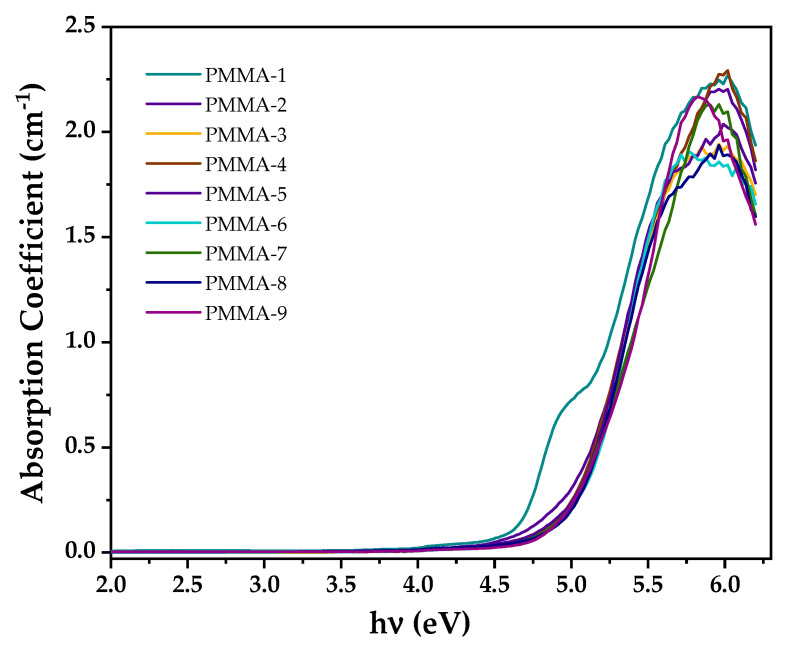
Variation of absorption coefficient (α) as a function of photon energy (hν) of PMMA microspheres.

To determine which transition type occurs in the PMMA microspheres, we used two approaches, according to Gupta et al. [36]; when the absorption coefficient α value is greater than $10^{4}\;{\mathrm{cm}}^{-1}$, electronic direct transition occurs, while, when $\alpha <10^{4}\;{\mathrm{cm}}^{-1}$, an indirect transition takes place. Figure 7 shows the absorption coefficient vs. incident radiation. It is observed that the absorption coefficient is less than $10^{4}\;{\mathrm{cm}}^{-1}$ in the energy range 4.5 to 5.7 eV; thus, we infer that the energy band transition in the PMMA under study is indirect. Additionally, the energy band structure of polymers has been investigated by calculating the Urbach energy using Equation (4):

(4) $$\alpha =\alpha_{0}exp\left(\frac{h\upsilon }{E_{t}}\right)$$

where $E_{t}$ is the Urbach energy tail.

It has been demonstrated that the $F\left(R_{\infty }\right)\sim \alpha$ approximation is considered precise enough to evaluate the optical properties of materials via specular reflectance [37]; therefore, Equation (4) is approximated to:

(5) $$F\left(R_{\infty }\right)=\alpha_{0}exp\left(\frac{h\upsilon }{E_{t}}\right)$$

where $F\left(R_{\infty }\right)$ is the optical absorption coefficient, $\alpha_{0}$ is a constant, hν is the incident photon energy, and $E_{t}$ is the Urbach energy that refers to the width of tails of the localized states. $E_{t}$ can be determined by taking the reciprocal slope of the straight line in the lnα vs. hν plot. The corresponding results are listed in Table 3. In this table, it is seen that the E_g_ values considering either a direct or an indirect energy band gap decrease as E_t_ values increase. Since the Urbach energy is the band tail of localized states in the energy band gap, it is expected that, in the PMMA, indirect transitions take place first; then, with the help of phonons, the transition to the conduction band occurs (LUMO). As a matter of fact, $\left(E_{g/I}+E_{t}\right)$ is approximately equal to $E_{g/D}$ [38].

### 3.2. PMMA Opals

**Figure 8:**
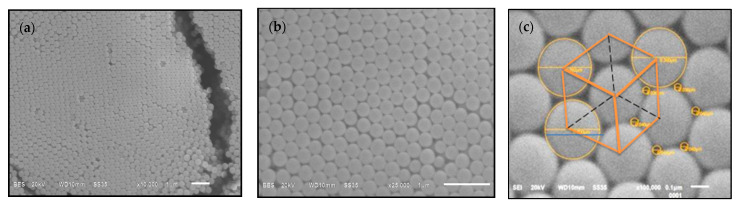
SEM images of PMMA opals prepared with PMMA_4 microspheres showing self-assembly of the microspheres: (**a**) 10,000×, (**b**) 25,000× and (**c**) 100,000×.

Figure 8 shows SEM micrographs of PMMA microspheres forming an opal or colloidal crystal. In (a) at ×25,000, we observe the self-organization properties and an intrinsic disorder that is inevitable in the self-ensemble process of colloidal spheres. In (b), some regions with continuous periodic array can be seen in more detail. In (c), on a SEM micrograph at ×100,000, a face center cubic (fcc) structure formed by the PMMA spheres with the (111) planes parallel to the subjacent substrate is delineated. This structure may not be a pure fcc with a repeated ABC stacking sequence along the growth direction [39] since, according to the studies of Versmold [40] and Verhaegh et al. [41], the most common type or structure in shear-ordered colloidal crystals is the close random stacking of close-packed planes.

**Figure 9:**
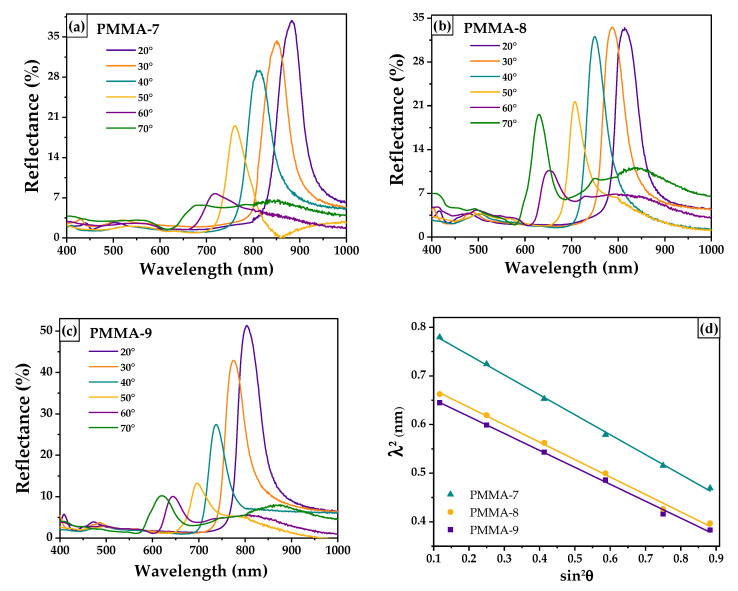
Specular reflectance spectra of the opals’ photonic crystals (**a**) PMMA-7, (**b**) PMMA-8, and (**c**) PMMA-9. In (**d**), the linear behavior of the stop bands’ angle dependent can be observed.

Figure 9a–c shows specular reflectance spectra of the opal photonic crystals vs. wavelength, measured at different incident angles (20° to 70°, $∆=10^{{}^{\circ}}$) and prepared with single microspheres labeled as PMMA-7, PMMA-8, and PMMA-9. Due to the high periodicity of PMMA microspheres that constitute each opal, they exhibit narrow photonic band gaps (PBGs) in the (111) direction.

The stop band position of the opal photonic crystal satisfices the Bragg–Snell law given by Equation (6), [1]:

(6) $$\lambda =2\left(d_{hkl}/m\right){\left(n_{eff}^{2}-sin^{2}\theta \right)}^{1/2}$$

where λ is the wavelength position of the Γ−L stop bands, $d_{hkl}$ is the distance between the *h, k, l* planes, m is the order of the Bragg diffraction, $n_{eff}$ is the effective refractive index of the material, and θ is the incident angle of light during measurements. The effective refractive index is calculated using Equation (7):

(7) $$n_{eff}=\varphi \times n_{\mathrm{PMMA}}+\left(1-\varphi \right)n_{air}$$

where φ is the solid volume fraction for *fcc*, the structure is φ=0.74, $n_{PMMA}$, and $n_{air}$, which are the refractive indices of the PMMA (1.492) and of air (1.000), then $n_{eff}=1.364$. In the case of first order diffraction from (111) planes, m=1 and $d_{hkl}=d_{111}=0.816D$, where D is the diameter of the PMMA microspheres. Then Equation (6) takes on the form of:

(8) $$\lambda =1.632D\sqrt[{}]{1.364^{2}-sin^{2}\theta }$$

From the $\lambda^{2}$ vs. $sin^{2}\theta$ plot shown in Figure 9d, it is seen that a Bragg diffraction condition is satisfied because this is a straight-line result; moreover, the slope is equal to $-{\left(2d_{111}\right)}^{2}$ and the y-axis intercept is ${\left(2d_{111}\right)}^{2}n_{eff}^{2}$. Then, from this relation, it was easy to calculate the interplanar distance ($d_{111})$, the effective refractive index ($n_{eff})$, the diameter of the PMMA microspheres (*D*), and the solid volume fraction (φ). These results are listed in Table 4.

Figure 9 shows the pseudo photonic band gap of each opal photonic crystal. These pseudo photonic band gaps are formed because the refractive indices ratio is less than 2.9, a required value to obtain a complete stop-band [1]. In a stop-band, all light is reflected and no propagated mode in the medium is found. In Figure 9a–c, it is seen that the positions of the pseudo stop-bands are shifted towards shorter wavelengths as θ increases according to Equation (8). 

The position of the stop bands at a 20° incident angle of the opals’ photonic crystals are: PMMA-7 at 883 nm, PMMA-8, 814 nm, and PMMA-9 at 803 nm. It is observed that the largest stop-band position corresponds to the sample with the largest diameter, PMMA-7 (323 nm). These results agree with those shown in Table 1, confirming an increment in size of the PMMA microspheres as the initiator amount increases. Furthermore, we can infer from these experimental results that the PBGs of the opals’ photonic crystals can be designed in accordance with the size of the PMMA microspheres throughout the optimization of initiator and monomer optimal amounts.

From the data in Table 4, we note that the calculated solid volume fractions exceed the theoretical value of 0.74 for an fcc sphere from hard materials. This is probably because PMMA spheres are considered soft material spheres [42].

## 4. Conclusions

PMMA microspheres were synthesized by means of the surfactant-free emulsion polymerization method by systematically varying the amount of initiator and the amount of monomer. The proportion of these precursors influences the diameter of PMMA microspheres. As the mmol of monomer and initiator increases, the microspheres’ size increases, except when a minimum quantity of monomer (190 mmol) is used, since the monomer acts as a limiting reactant. For this particular monomer quantity, the PMMA microspheres’ size remains practically constant. The obtained colloidal microspheres are highly stable and present a surface with positive charge due to the protonation of the carboxyl group of the PMMA. The microspheres under study present an energy band gap less than the theoretical one reported in the literature, since PMMA microspheres are porous and present punctual and structural defects. The E_g_ values were calculated considering both direct and indirect energy band structures. The energy band gap and the Urbach energy values of PMMA allowed us to conclude that that PMMA energy band gap structure was indirect.

All synthesized opal photonic crystals present narrow stop bands since the refractive index contrast of the two media is less than the minimum required (2.9). The stop bands shift towards larger wavelengths as the microsphere size increases. It was also observed that PBGs shifted towards shorter wavelengths and the intensity decreased as the incident angle of irradiation increased. The Bragg–Snell law is satisfied since the diffraction and the reflection effects take place in the opal photonic crystal.

## Figures and Tables

**Table 1:** Synthesis conditions of PMMA microspheres, average diameter measured by SEM (D_avg_), hydrodynamic diameter measured by DLS system (D_h_), PDI and Zeta Potential values (ζ ).

Sample	Monomer (mmol)	Initiator (mmol)	$D_{avg}\;(\mathrm{nm})$	$D_{h}\;(\mathrm{nm})$	PDI	ζ (mV)
PMMA-1	190	1.65	235 ± 9.0	292 ± 17.1	0.034	47.3 ± 5.3
PMMA-2	190	1.10	244 ± 11.2	301 ± 17.3	0.023	43.9 ± 5.2
PMMA-3	190	0.55	247 ± 15.1	303 ± 17.4	0.069	47.2 ± 5.5
PMMA-4	285	1.65	298 ± 8.0	390 ± 19.7	0.045	51.1 ± 5.4
PMMA-5	285	1.10	273 ± 7.0	380 ± 19.5	0.034	48.5 ± 5.7
PMMA-6	285	0.55	249 ± 7.0	360 ± 19.0	0.042	49.7 ± 5.4
PMMA-7	380	1.65	323 ± 9.0	413 ± 20.3	0.083	46.1 ± 4.9
PMMA-8	380	1.10	311 ± 8.1	398 ± 20.0	0.082	41.4 ± 4.4
PMMA-9	380	0.55	306 ± 11.0	379 ± 19.5	0.088	46.1 ± 4.8

**Table 2:** Effect of the variation of monomer and initiator in the synthesis of various polymers using the SFEP method.

Polymer	Initiator	Monomer	T (℃)	D_avg_ Trend Monomer	D_avg_ Trend Initiator	Reference
PMS	V-50 4.23 mM 22.3 mM	Methylstyrene 424 mM	70		It decreases with increasing initiator concentration.	[19]
PMMA	V-50 0.75 to 3.0 g	Methyl methacrylate 300 to 400 mL	70 to 80	D_avg_ increases from 364 to 415 nm when monomer amount increases from 300 to 400 mL at fixed 1.5 g of initiator and T = 70 ℃**.**	The effect of initiator amount is not conclusive because amount of monomer and temperature is varied simultaneously.	[1]
PMMA	V-50 0.034, 0.061, 0.186 g	Methyl methacrylate 28.3, 14.2, 2.83 g	70		For fixed monomer amounts: 28.3, 14.2 g, with increasing initiator, D_avg_ increases. For fixed monomer amount: 2.83 g, with increasing initiator, D_avg_ decreases.	[20]
PS	AIBA 0.0008, 0.004, 0.008, 0.04 wt%	Styrene 0.40, 0.80, 2.0 wt%	55 to 90	For each amount of initiator, the monomer was varied to three quantities. A similar trend was observed: the D_avg_ was increased as the monomer amount was increased.	For each amount of monomer, the initiator was varied to four quantities. A similar trend was observed: the D_av_*_g_* was increased as the initiator amount was increased.	[6]
PS	KPS 0.05, 0.2 g	Styrene 5%, 10%, 14% (*v*/*v*)	80	The monomer was varied to three quantities. The D_avg_ of polystyrene spheres increased 223 to 316 nm when styrene monomer concentration was increased.	When the styrene monomer concentration was set constant at 10% (*v*/*v*), the D_avg_ of the PS particles decreased from 249 nm using 0.05 g of initiator, to 181 nm using 0.2 g of initiator.	[21]
PMMA	V-50 0.55, 1.10, 1.65 mmol	Methyl methacrylate 190, 285, 380 mmol	75	For each amount of initiator, the monomer was varied to three quantities. In all cases a similar non-linear trend was observed: the D_avg_ is increased as the monomer amount is increased.	For fixed monomer amounts: 380, 285 mmol, with increasing initiator, D_avg_ increased. For fixed monomer amount: 190 g, with increasing initiator, D_avg_ decreased.	This work

PMS: poly (methyl styrene); PS: poly (styrene); PMMA: poly (methyl methacrylate).

**Table 3:** Direct energy band gap (E_g/D_), indirect energy band gap (E_g/I_), Urbach energy (E_t_), and absorption constant $\left(\alpha_{0}\right)$ of the obtained PMMA microspheres.

Sample	E_g/D_ (eV)	E_g/I_ (eV)	$E_{t}\;(\mathrm{eV})$	$\alpha_{0}\;10^{-5}\;({\mathrm{cm}}^{-1})$
PMMA-1	5.23 ± 0.06	4.68 ± 0.01	0.484	2.030
PMMA-2	5.22 ± 0.05	4.54 ± 0.03	0.509	2.870
PMMA-3	5.15 ± 0.06	4.36 ± 0.02	0.558	3.500
PMMA-4	5.19 ±0.07	4.65 ± 0.05	0.522	3.920
PMMA-5	5.22 ± 0.09	4.71 ± 0.04	0.403	2.670
PMMA-6	5.23 ± 0.06	4.75 ± 0.03	0.479	1.500
PMMA-7	5.21 ± 0.04	4.61 ± 0.02	0.544	5.089
PMMA-8	5.34 ± 0.05	4.65 ± 0.03	0.537	4.992
PMMA-9	5.35 ± 0.06	4.73 ± 0.03	0.403	0.155

**Table 4:** Wavelength position of opals’ stop band (λ), intercept and slope fit data, interplanar distance (*d*_111_). diameter of the PMMA microspheres (*D*), and the solid volume fraction (φ) of the obtained opals’ photonic crystals.

Sample	λ (θ$=20^{{}^{\circ}})$ ^a^ (nm)	Intercept	Slope	*d*_111_^b^ (nm)	*D* ^b^ (nm)	$n_{eff}{\;}^{b}$	$\varphi {\;}^{b}$	$\lambda_{max}$(θ$=20^{{}^{\circ}})$ ^b^ (nm)
PMMA-7	883	0.8248	−0.4094	320 ± 22	392 ± 9	1.420 ± 0.02	0.852	880 ± 14
PMMA-8	814	0.7072	−0.3586	300 ± 18	367 ± 11	1.404 ± 0.01	0.820	816 ± 18
PMMA-9	803	0.6861	−0.3482	295 ± 15	361 ± 7	1.403 ± 0.03	0.820	803 ± 22

^a^ Data obtained directly from specular reflectance spectra. ^b^ Data calculated from Bragg–Snell law.

## Data Availability

The data presented in this study are available on request from the corresponding author.
